# Schisandrin A alleviates allergic rhinitis by restoring Th1/Th2 and Treg/Th17 immune balance

**DOI:** 10.1515/biol-2025-1348

**Published:** 2026-08-25

**Authors:** Zhipeng Zhang, Shan He, Yanmin Yu, Yongjun Deng, Yuan Chen, Xiaoyi Gu, Xiaoyi Qiu, Jingjing Wei, Yan Shi, Yu Jiao

**Affiliations:** Department of Otolaryngology, Shanghai Pudong New Area Hospital of Traditional Chinese Medicine, Shanghai, China; Department of Otolaryngology, Shanghai Children’s Medical Center, Shanghai Jiao Tong University School of Medicine, Shanghai, 200120, China; Department of Otolaryngology, Shanghai Pudong New Area Guangming Hospital of Traditional Chinese Medicine, Shanghai, China

**Keywords:** Schisandrin A, allergic rhinitis, Treg, Th17, PI3K/AKT

## Abstract

Allergic rhinitis (AR) is a common chronic inflammatory disorder characterized by nasal mucosal inflammation, sneezing, and rhinorrhea. Schisandrin A (Schis-A) has demonstrated significant anti-inflammatory effects in models of allergic asthma. This study aimed to evaluate the therapeutic efficacy of Schis-A in AR and to elucidate its underlying molecular mechanisms. An AR mouse model was established by sensitization and challenge with ovalbumin (OVA), followed by oral administration of Schis-A at doses of 40 and 80 mg/kg/day. Nasal symptoms were monitored and quantified. OVA-specific immunoglobulin E (IgE) levels and T helper (Th)1/Th2- and regulatory T cells (Treg)/Th17-associated cytokines were measured using enzyme-linked immunosorbent assay and quantitative real-time PCR. Treg and Th17 cell populations were analyzed by flow cytometry. The results showed that Schis-A treatment significantly reduced OVA-induced nasal rubbing and sneezing, indicating effective attenuation of allergic responses. Serum OVA-specific IgE levels were markedly reduced in Schis-A-treated AR mice. Moreover, Schis-A restored Th1/Th2 and Treg/Th17 cytokine balance, as indicated by upregulated expression of IFN-γ, IL-2, and Foxp3, alongside downregulation of IL-4, IL-5, IL-13, and IL-17. Flow cytometric analysis confirmed an increase in Treg cells and a reduction in Th17 cell populations. Mechanistically, Schis-A inhibited activation of the PI3K/AKT signaling pathway, suggesting that this pathway may contribute to its immunomodulatory effects. Schis-A alleviates AR by modulating Th1/Th2 and Treg/Th17 immune responses and inhibiting PI3K/AKT pathway activation, offering a promising therapeutic strategy for the treatment of AR.

## 1 Introduction

Allergic rhinitis (AR) is a global public health concern affecting approximately 10–14 % of the population [1]. In high-income countries, the prevalence of this chronic condition can reach up to 50 %, whereas its incidence remains relatively low in middle- and low-income regions [2], 3], although the prevalence is steadily increasing. AR is an IgE-mediated hypersensitivity reaction triggered by exposure to allergens such as dust mites, pet dander, and pollen [3], 4]. Clinical symptoms, such as sneezing, rhinorrhea, nasal pruritus, and nasal congestion, significantly compromise the patient’s quality of life. Current therapeutic options, including antihistamines, decongestants, and immunotherapy [5], 6], primarily target symptom relief but fail to address the underlying immunological dysregulation.

Dysregulated immune responses, especially the imbalance between T helper (Th) cell subsets, are key drivers of AR pathogenesis [7], 8]. A shift toward Th2 polarization plays a central role in AR pathogenesis, characterized by elevated levels of Th2 cytokines such as interleukin (IL)-4, IL-5, and IL-13, which promote immunoglobulin E (IgE) production and mast cell sensitization [9]. In contrast, Th1 cells secrete interferon gamma (IFN-γ), which suppresses Th2-mediated responses and contributes to immune homeostasis [10]. Beyond the Th1/Th2 axis, emerging evidence highlights the involvement of regulatory T cells (Treg) and Th17 cells in AR [11], 12]. Treg cells maintain immune tolerance and inhibit excessive inflammation [13], whereas Th17 cells promote pro-inflammatory cytokine release and are associated with eosinophilic and neutrophilic infiltration. Patients with AR often exhibit a disrupted Treg/Th17 balance, marked by reduced Treg activity and enhanced Th17 responses, which exacerbates mucosal inflammation [14]. Therefore, restoring Th1/Th2 and Treg/Th17 balance is considered a promising immunomodulatory strategy for AR.

The phosphatidylinositol 3-kinase/protein kinase B (PI3K/AKT) signaling pathway is widely recognized as a central regulatory axis involved in multiple biological processes, including cell proliferation, metabolism, and immune responses [15], 16]. Recent studies have demonstrated that aberrant activation of the PI3K/AKT signaling pathway contributes to the dysregulation of T cell subsets in allergic diseases [17], 18]. Specifically, PI3K/AKT signaling promotes Th2 and Th17 differentiation while suppressing Treg development, thereby exacerbating immune imbalance and inflammation [17], 19], 20]. Targeting this pathway may therefore offer a mechanistic basis for restoring Th1/Th2 and Treg/Th17 equilibrium.

Schisandrin A (Schis-A) is a bioactive lignan extracted from *Schisandra chinensis*, a traditional Chinese medicinal herb known for its broad pharmacological properties [21], 22]. Extensive studies have demonstrated that Schis-A exerts protective effects in various disease models, including asthma, neurodegenerative disorders, and diabetic nephropathy, primarily through its anti-inflammatory and antioxidant activities [23], [24], [25], [26]. Mechanistically, Schis-A has been shown to modulate multiple signaling pathways, notably the PI3K/AKT axis, which plays a critical role in regulating immune cell differentiation and inflammatory responses [27], 28]. Although Schis-A has demonstrated efficacy in alleviating airway inflammation in asthma models [26], its role in allergic rhinitis remains largely unexplored. Therefore, this study aims to investigate the therapeutic potential of Schis-A in allergic rhinitis and to elucidate its underlying immunomodulatory mechanisms, with a particular focus on Th1/Th2 and Treg/Th17 balance and PI3K/AKT signaling.

## 2 Materials and methods

### 2.1 Animals

All experimental procedures were performed in compliance with the ARRIVE guidelines to minimize animal suffering. Male BALB/c mice (8 weeks old, 20–25 g) were obtained from the Zhejiang Medical University Laboratory Animal Research Center (Zhejiang, China). All animals were housed in a specific pathogen-free (SPF) laboratory environment with controlled temperature (23–25 °C), humidity (50–60 %), and a 12-h light/dark cycle.

**Ethical approval:** The research related to animal use has been complied with all the relevant national regulations and institutional policies for the care and use of animals, and has been approved by the Animal Ethics Committee of Shanghai Pudong New Area Hospital of Traditional Chinese Medicine (No. pdzyyy-2022-06).

### 2.2 AR model establishment

The AR model was established according to previously described protocols [29] with minor modifications. Briefly, mice were sensitized intraperitoneally with 50 μg OVA (Sigma-Aldrich, MO, USA) emulsified in 25 μL Imject Alum (Thermo Fisher Scientific, MA, USA) on days 0, 7, and 14. From days 22–29, mice were challenged intranasally with 20 μL of 60 mg/mL OVA solution once daily. Mice were randomly assigned to five groups (*n* = 6 per group): (i) Control group: saline sensitization and challenge, (ii) AR model group: OVA sensitization and challenge, (iii) Schis-A low-dose group: OVA + Schis-A (40 mg/kg/day), (iv) Schis-A high-dose group: OVA + Schis-A (80 mg/kg/day), (v) Positive control group: OVA + Dexa (2 mg/kg/day). Schis-A (purity ≥98 %, MedChemExpress) was stored as a concentrated stock in 100 % DMSO at −80 °C. Working solutions (10 % DMSO in saline) were prepared fresh daily immediately before oral gavage to ensure stability and potency, consistent with manufacturer specifications and established protocols [26], 27]. Schis-A was administered via oral gavage once daily from day 20 to day 29. Dexa (Sigma-Aldrich) was similarly administered at 2 mg/kg/day during the same period.

### 2.3 Symptom scoring

Ten minutes after the final OVA challenge, two blinded observers recorded the number of sneezes and nasal rubs over a 20-min period to quantify allergic symptoms.

### 2.4 Enzyme-linked immunosorbent assay (ELISA) for cytokines and OVA-specific IgE

Whole blood was collected from mice, and serum was subsequently separated by centrifugation at 14,000 rpm for 15 min at 4 °C. Serum concentrations of IFN-γ (ab282874, Abcam, CA, USA), IL-2 (ab100706), IL-4 (ab100710), IL-5 (ab204523), IL-13 (ab219634), IL-10 (ab255729), and IL-17 (ab199081) were measured using commercial ELISA kits. OVA-specific IgE was quantified using mouse-specific ELISA kits (Beyotime, Shanghai, China). All assays were performed in duplicate according to the manufacturer’s instructions.

### 2.5 Flow cytometry

Mononuclear cells were obtained from spleens by Ficoll-Hypaque density gradient centrifugation [30] and cultured in RPMI-1640 medium containing 10 % FBS (GIBCO, NY, USA) and 1 % Penicillin-Streptomycin (Beyotime). Subsequently, cells were stimulated with PMA (50 ng/mL), ionomycin (500 ng/mL), and brefeldin A (all obtained from Sigma-Aldrich) for 5 h prior to conducting FACS analysis. For Th17 (CD4^+^ IL-17a^+^) cell detection, 1 × 10^6^ mononuclear cells were labeled with CD4-FITC antibody (ab269349, Abcam) for 30 min at 4 °C. Cells were then fixed and permeabilized with a fix/perm solution and stained using an IL-17a-PE antibody (12-7177-81, Thermo Fisher Scientific) for 30 min at 4 °C. For Treg (CD4^+^CD25^+^Foxp3^+^) cell detection, 1 × 10^6^ mononuclear cells were co-stained with CD4-FITC (ab269349) and CD25-APC antibodies (17-0251-82, Thermo Fisher Scientific) for 30 min at 4 °C. The cells were then fixed and permeabilized with a fix/perm solution (Sigma-Aldrich) as per the manufacturer’s protocol and labeled with Foxp3-PE antibody (ab218773, Abcam) for 40 min at 4 °C. An Attune NxT flow cytometer (Thermo Fisher Scientific) was used to assess Th17 and Treg cell percentages, and FlowJo software was used for data analysis.

### 2.6 Quantitative real-time PCR (qRT-PCR)

Total RNA from nasal tissues was extracted using TRIzol reagent (Invitrogen), and cDNA was synthesized using a reverse transcription kit (Takara, Japan). qRT-PCR was performed using SYBR Green Master Mix (Bio-Rad) on a CFX96 system (Thermo Fisher Scientific). Relative gene expression of Foxp3 and RORγt was normalized to β-actin using the 2^−ΔΔCt^ method [31]. All primer sequences used for qRT-PCR were detailed in Supplementary Table S1.

### 2.7 Western blotting

Total proteins were isolated from nasal mucosa using RIPA buffer (Beyotime, Shanghai, China), quantified by the BCA method (Beyotime), and separated by 10 % SDS-PAGE. Subsequently, proteins were electrotransferred onto PVDF membranes (Roche, Basel, Switzerland). After blocking with 3 % BSA for 1 h at room temperature, membranes were incubated for 2 h at room temperature with the primary antibodies against PI3K (ab302958, Abcam), phospho-PI3K (p-PI3K, ab182651, Abcam), Akt (ab8805, Abcam), p-Akt (ab38449, Abcam), and β-actin (ab8226, Abcam). After washing with TBST buffer (Beyotime), membranes were incubated with goat anti-rabbit secondary antibody (A27036, Thermo Fisher Scientific) and then visualized with an ECL reagent (Beyotime).

### 2.8 Hematoxylin and eosin (H&E) and periodic acid–Schiff (PAS) staining

Paraffin-embedded nasal tissues were sectioned at a thickness of 5 μm, deparaffinized, and rehydrated. The sections were then stained with H&E and PAS staining kits (Beyotime), and images were captured using an N-800M light microscope (OLABO, Shangdong, China).

### 2.9 Statistics

Results represent the mean ± SD from a minimum of three independent experiments. Statistical comparisons among multiple groups were performed using one-way ANOVA followed by Scheffé’s post hoc test in GraphPad Prism 7.0 (GraphPad Software Inc., CA, USA). Differences were considered statistically significant at *p* < 0.05.

## 3 Results

### 3.1 Schis-A alleviated allergic symptoms in AR mice

**Figure 1:**
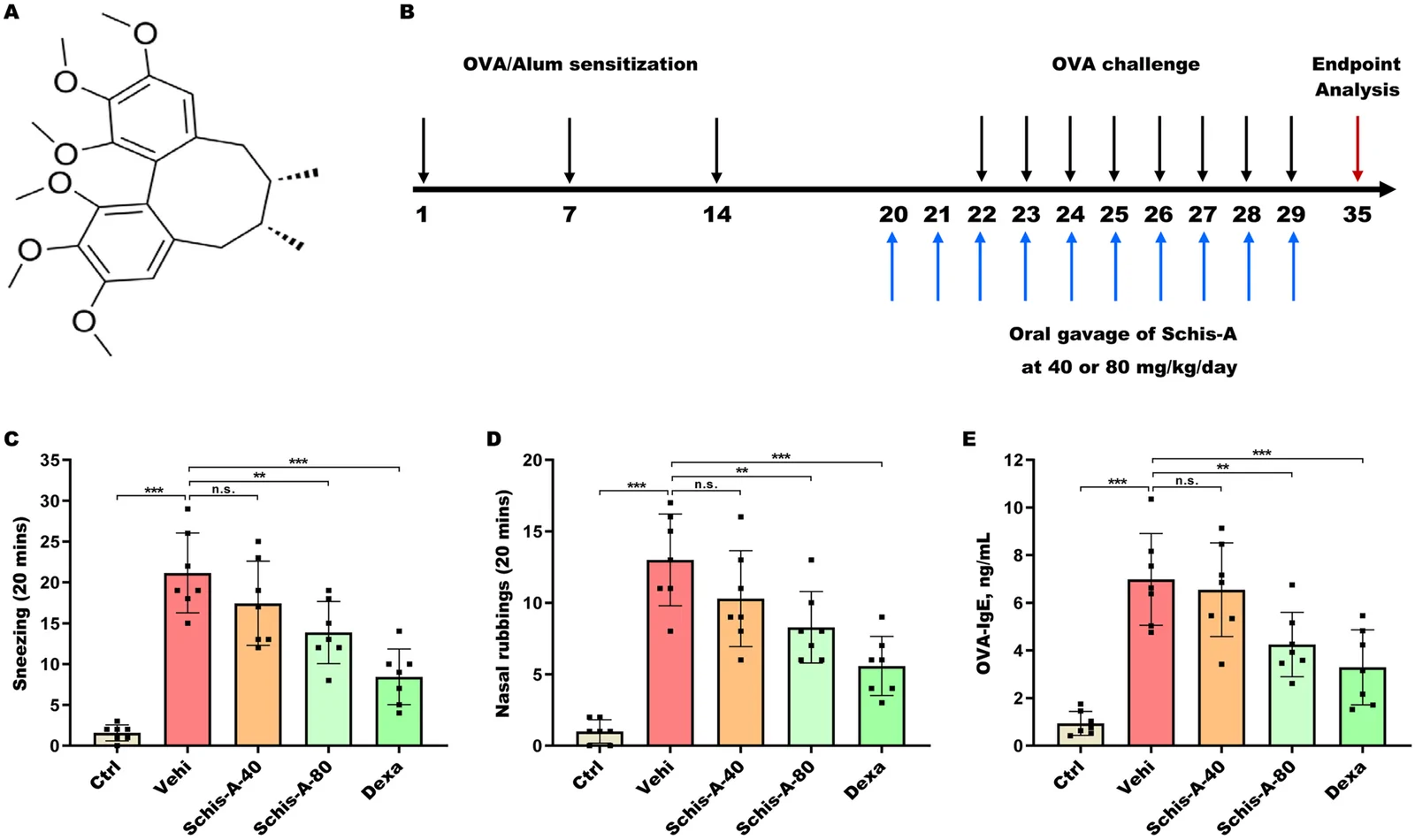
Schis-A alleviated allergic symptoms in AR mice. (A) Chemical structure of Schis-A. (B) Experimental timeline showing OVA sensitization on days 0, 7, and 14, followed by intranasal challenge from days 22–29. Schis-A (40 or 80 mg/kg) and Dexa (2 mg/kg) were administered orally once daily during the challenge phase. (C and D) Quantification of sneezing and nasal rubbing frequencies recorded over a 20-min period following the final OVA challenge, reflecting nasal hypersensitivity. (E) Serum levels of OVA-specific IgE measured by ELISA, indicating systemic allergic sensitization. Data are presented as mean ± SD (*n* = 6 per group). *p < 0.05, **p < 0.01, ***p < 0.001 versus vehicle group. *n*.s., not significant.

To evaluate the therapeutic effects of Schis-A in an OVA-induced AR mouse model, we assessed nasal symptom behaviors and serum IgE levels. Behavioral observations over a 20-min period revealed that OVA challenge significantly increased the frequency of sneezing and nasal rubbing compared to the control group, reflecting increased nasal irritation and allergic responses. Mice treated with Schis-A exhibited a dose-dependent trend in both sneezing (Figure 1C) and nasal rubbing (Figure 1D), suggesting that Schis-A effectively suppresses allergen-induced nasal hypersensitivity.

Serological analysis showed that OVA-specific IgE levels were markedly elevated in vehicle-treated AR mice, confirming successful sensitization and Th2-skewed humoral activation (Figure 1E). Schis-A treatment significantly reduced serum IgE concentrations in a dose-dependent trend, with both 40 and 80 mg/kg groups showing clear suppression relative to vehicle controls.

**Figure 2:**
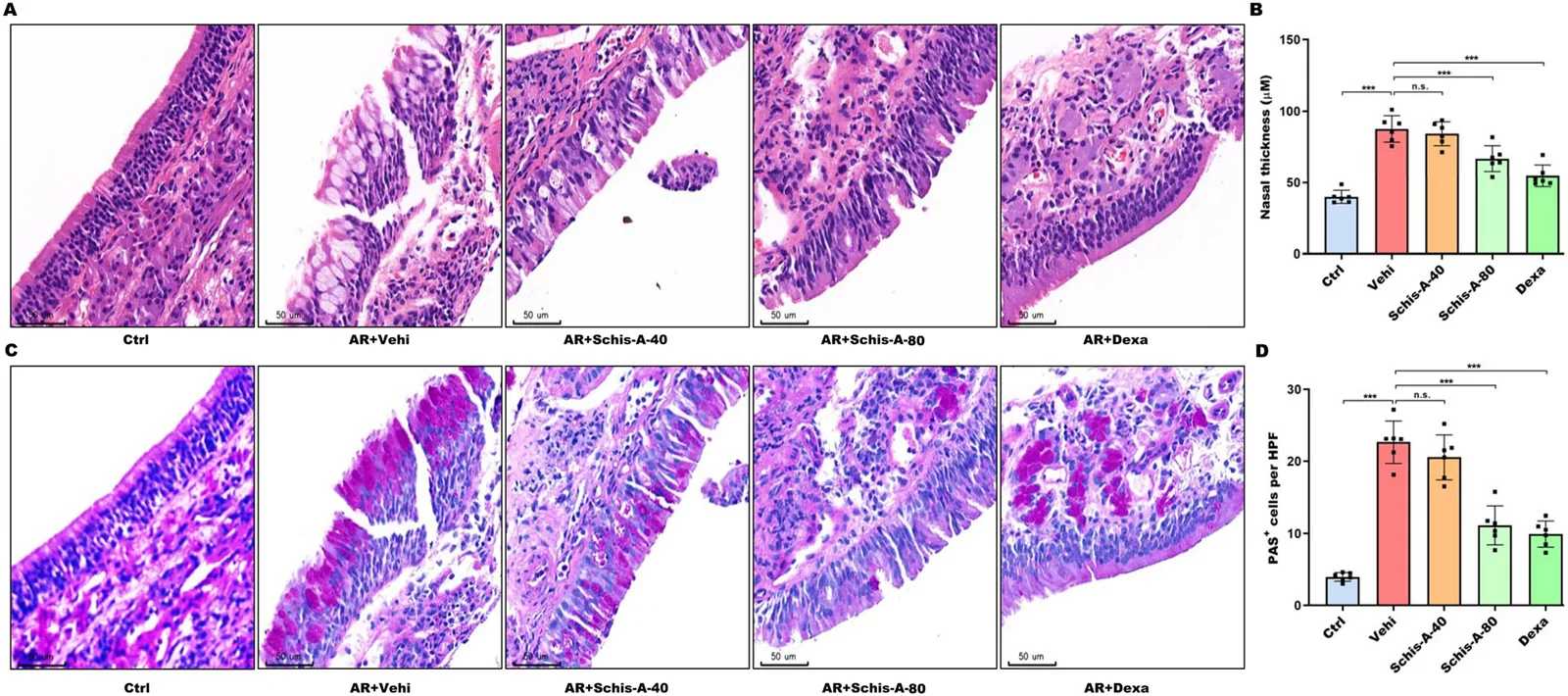
Histological effects of Schis-A on nasal tissues. Representative H&E (A) and PAS (C) staining images of nasal sections from the control (Ctrl), AR model (vehicle), Schis-A-treated (40 or 80 mg/kg), and Dexa groups. Quantitative analyses of epithelial thickness (B) and goblet cell hyperplasia (D) were performed for the same groups. ***p < 0.001 versus vehicle group.

The histopathological effects of Schis-A on nasal mucosa were evaluated. In control mice, H&E staining showed well-organized epithelial and ciliated structures, whereas AR mice exhibited epithelial desquamation and vascular dilation within the lamina propria (Figure 2A). Treatment with Schis-A markedly alleviated these lesions. The pronounced epithelial thickening observed in AR mice was significantly reduced following Schis-A administration (Figure 2B). Likewise, Schis-A attenuated the extensive goblet cell hyperplasia evident in the nasal mucosa of AR mice (Figure 2C and D). These findings suggest that Schis-A not only alleviates behavioral symptoms of AR but also attenuates underlying immunological sensitization.

### 3.2 Schis-A restored the Th1/Th2 cytokine balance in AR mice

**Figure 3:**
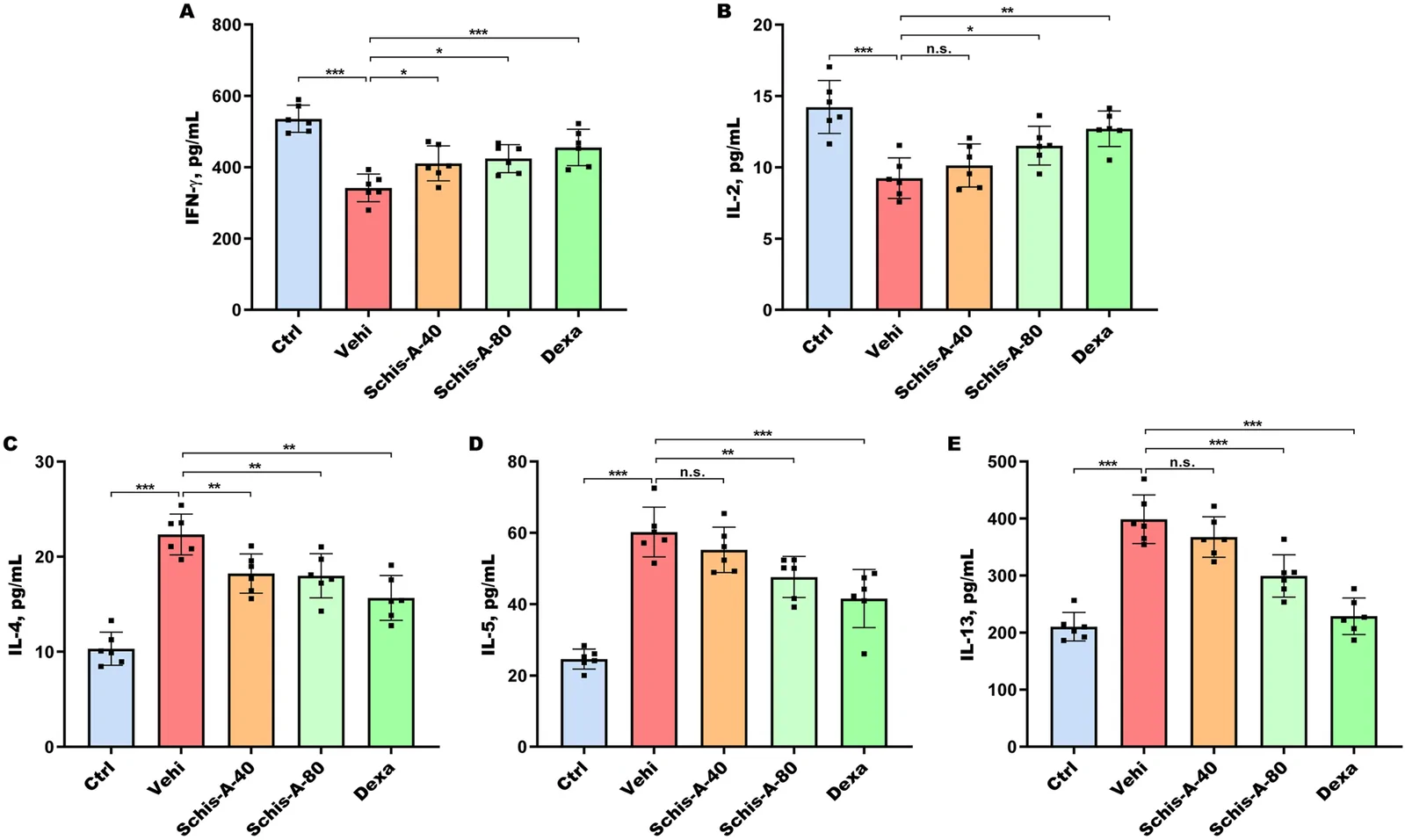
Schis-A restored Th1/Th2 cytokine balance in AR mice. Blood samples were collected at the end of the experiment. Cytokine concentrations were determined using commercial ELISA kits. (A and B) Serum levels of Th1 cytokines IFN-γ and IL-2. (C–E) Serum levels of Th2 cytokines IL-4, IL-5, and IL-13. Data are presented as mean ± SD (*n* = 6 per group). *p < 0.05, **p < 0.01, ***p < 0.001 versus vehicle group. *n*.s., not significant.

To evaluate the immunomodulatory effects of Schis-A on Th1/Th2 polarization in AR, we quantified serum levels of key cytokines. As shown in Figure 3A and B, vehicle-treated AR mice exhibited a marked reduction in Th1-associated cytokines IFN-γ and IL-2 compared to control mice, indicating a shift toward Th2 dominance. Schis-A treatment reversed this trend in a dose-dependent manner, with the 80 mg/kg group showing substantial restoration of Th1 cytokine levels.

In contrast, Th2 cytokines, including IL-4, IL-5, and IL-13, were significantly elevated in the vehicle group (Figure 3C–E), consistent with enhanced humoral activation and allergic inflammation. Administration of Schis-A suppressed all three Th2 cytokines, with the higher dose achieving reductions comparable to those observed in the Dexa group. These results suggest that Schis-A effectively rebalances Th1/Th2 immune responses by promoting Th1 cytokine expression while attenuating Th2-mediated inflammation.

### 3.3 Schis-A restored Treg/Th17 immune balance in AR mice

**Figure 4:**
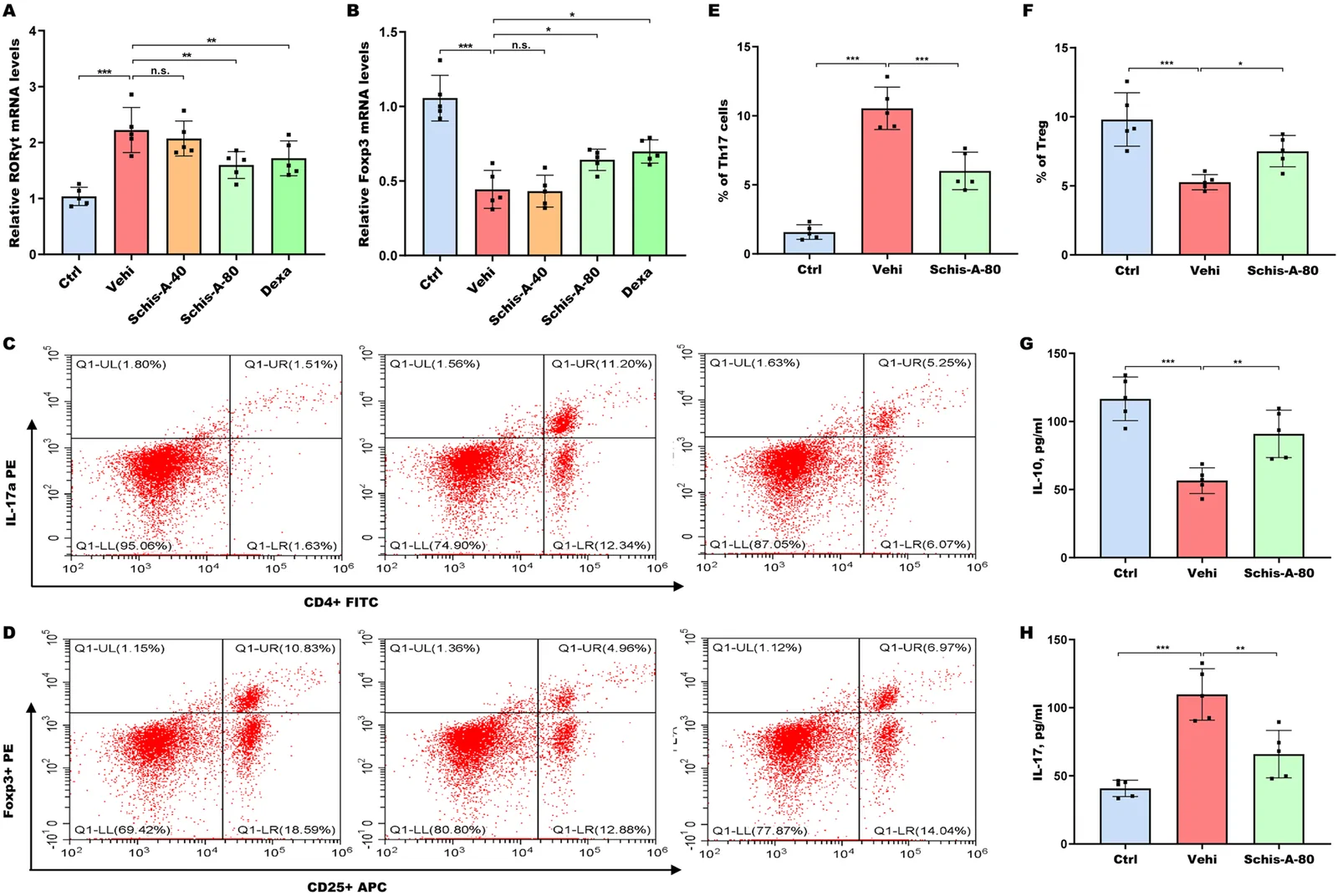
Schis-A restored Treg/Th17 immune balance in AR mice. Nasal tissues and blood samples were collected at the end of the experiment. (A and B) Relative mRNA levels of RORγt and Foxp3 in nasal tissues were measured by qRT-PCR. (C and D) Flow cytometry plots showing CD4^+^ IL-17A^+^ Th17 cells and CD25^+^ Foxp3^+^ Tregs. (E and F) Quantification of Th17 and Treg cell percentages. (G and H) Serum levels of IL-10 and IL-17 were measured by ELISA. Data are presented as mean ± SD (*n* = 5 per group). *p < 0.05, **p < 0.01, ***p < 0.001 versus vehicle group. *n*.s., not significant.

To investigate the effects of Schis-A on Treg/Th17 dysregulation in AR, we assessed transcriptional, cellular, and cytokine-level indicators of immune polarization. As shown in Figure 4A and B, vehicle-treated AR mice exhibited elevated expression of retinoic acid-related orphan receptor gamma t (RORγt) and reduced expression of Forkhead box protein P3 (Foxp3), reflecting a shift toward Th17 dominance and impaired Treg differentiation. Schis-A treatment reversed this transcriptional imbalance, with the 80 mg/kg group showing normalization comparable to Dexa, whereas the 40 mg/kg group did not exhibit statistically significant changes.

Flow cytometry analysis further confirmed these findings. The proportion of CD4^+^ IL-17A^+^ Th17 cells was markedly increased in AR mice (Figure 4C–E), accompanied by a reduction in CD25^+^ Foxp3^+^ regulatory T cells (Figure 4D–F), indicating a disrupted Treg/Th17 balance. Schis-A administration reversed this pattern, significantly reducing Th17 cell frequency and restoring Treg proportions in a dose-dependent manner.

Cytokine profiling supported these cellular changes. IL-10, a key anti-inflammatory cytokine produced by Tregs, was significantly suppressed in AR mice (Figure 4G), while IL-17 levels were markedly elevated (Figure 4H). Schis-A treatment normalized both cytokines, reinforcing its role in reestablishing immune homeostasis through dual modulation of proinflammatory and regulatory pathways.

### 3.4 Schis-A suppressed PI3K/AKT signaling activation in AR

**Figure 5:**
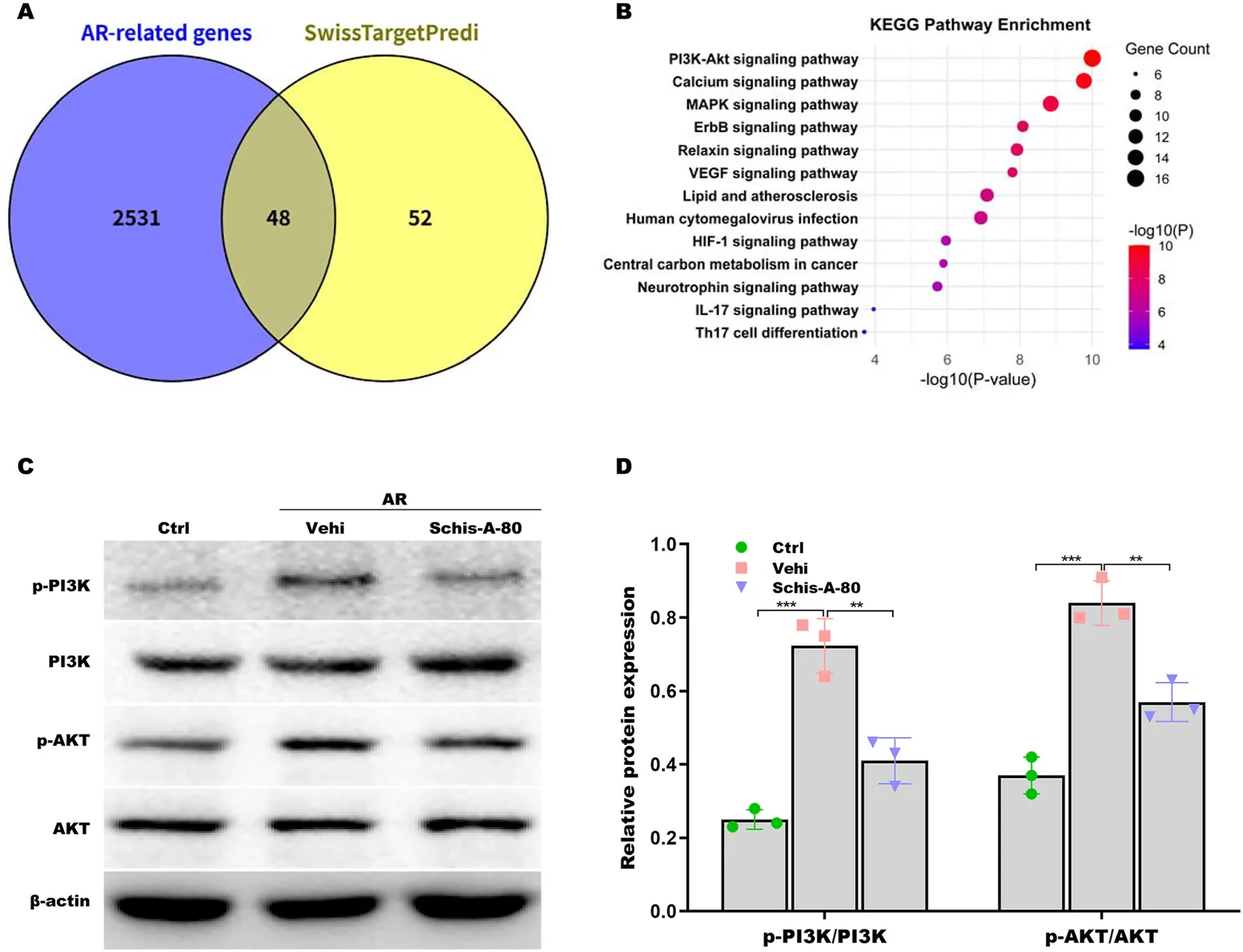
Schis-A suppressed PI3K/AKT signaling activation in AR. (A) Venn diagram showing overlap between AR-related genes (GeneCards) and Schis-A predicted targets (SwissTargetPrediction), identifying 48 shared genes. (B) KEGG pathway enrichment analysis of overlapping genes. (C) Western blot analysis of total and phosphorylated PI3K and AKT proteins in nasal tissues from control, AR, and Schis-A (80 mg/kg) groups. (D) Quantification of p-PI3K/PI3K and p-AKT/AKT ratios. Nasal tissues were collected at the end of the experiment. Data are presented as mean ± SD (*n* = 5 per group). **p < 0.01, ***p < 0.001.

To elucidate the molecular mechanisms underlying the therapeutic effects of Schis-A in AR, potential targets were identified using the SwissTargetPrediction database, yielding 100 candidate genes (Supplementary Table S2). In parallel, 2,479 AR-associated genes were retrieved from the GeneCards database (Supplementary Table S3). Intersection analysis revealed 48 overlapping genes (Supplementary Table S4; Figure 5A), representing a subset of biologically relevant targets. These genes were subsequently subjected to KEGG pathway enrichment to identify signaling cascades potentially modulated by Schis-A.

Enrichment analysis based on KEGG demonstrated a prominent involvement of the PI3K–Akt signaling, along with other inflammation-related cascades such as calcium signaling, mitogen-activated protein kinase (MAPK), and tumor necrosis factor (TNF) pathways (Figure 5B). To experimentally validate these predictions, we assessed the expression of PI3K and AKT, along with their phosphorylated forms, in nasal tissues. As shown in Figure 5C, AR mice exhibited elevated levels of p-PI3K and p-AKT, indicating activation of the PI3K/AKT axis. Treatment with Schis-A (80 mg/kg) markedly reduced phosphorylation of both proteins, while total PI3K and AKT levels remained unchanged (Figure 5D). These findings suggest that Schis-A exerts its therapeutic effects, at least in part, by suppressing PI3K/AKT signaling, thereby contributing to the restoration of Th1/Th2 and Treg/Th17 immune balance.

## 4 Discussion

This study demonstrates that Schis-A effectively alleviates AR symptoms in an OVA-induced mouse model. Schis-A reduced sneezing and nasal rubbing behaviors, suppressed serum OVA-specific IgE levels, and restored Th1/Th2 and Treg/Th17 immune balance. Additionally, Schis-A inhibited phosphorylation of PI3K and AKT in nasal tissues, suggesting a mechanistic role for PI3K/AKT signaling in its immunomodulatory effects.

Recent studies have increasingly highlighted that immune dysregulation in AR extends beyond the classical Th1/Th2 paradigm to include Treg/Th17 imbalance, epithelial barrier dysfunction, and metabolic reprogramming [32], [33], [34]. Specifically, Th17 cell expansion and impaired Treg activity drive chronic inflammation. This is initiated by compromised epithelial tight junctions, which release alarmins like TSLP (thymic stromal lymphopoietin) and IL-33 to amplify systemic inflammation [33]. Furthermore, microbiota-influenced metabolic shifts, such as malate levels, further regulate T-cell differentiation, sustaining the systemic allergic state [32], 35]. The observed rebalancing of Th1/Th2 and Treg/Th17 subsets is particularly relevant, given their central role in AR pathogenesis [36], [37], [38]. Th2 and Th17 dominance, coupled with impaired Th1 and Treg responses, drives IgE production, eosinophilic infiltration, and mucosal inflammation [39], [40], [41], [42]. By promoting IFN-γ, IL-2, and Foxp3 expression while suppressing IL-4, IL-5, IL-13, and IL-17, Schis-A appears to shift the immune landscape toward resolution. However, whether this shift is a direct consequence of PI3K/AKT inhibition remains uncertain. It remains unclear whether Schis-A acts through upstream regulators of T cell differentiation, warranting further investigation.

The broader therapeutic implications of Schis-A are also worth considering. Previous studies in asthma models have shown similar immunoregulatory effects [26], suggesting that Schis-A may exert conserved actions across allergic airway diseases. Moreover, its ability to modulate multiple immune axes raises the possibility of synergistic effects when combined with existing therapies [27], 43]. Yet, the specificity of Schis-A’s effects on distinct T cell subsets and its potential off-target actions remain open questions. For instance, could its antioxidant properties indirectly shape cytokine profiles by modulating oxidative stress in the nasal microenvironment? These possibilities highlight the need for more granular cellular and molecular analyses.

Another point of interest is the pharmacological behavior of Schis-A itself. While its efficacy in modulating immune responses is evident, little is known about its bioavailability, tissue distribution, or metabolic stability *in vivo*. Does Schis-A accumulate preferentially in mucosal tissues, or is its effect systemic? Understanding its pharmacokinetics could inform dosing strategies and delivery methods, especially for chronic allergic conditions requiring sustained immunomodulation.

Despite these promising findings, several limitations should be acknowledged. First, although Schis-A improved Th1/Th2 and Treg/Th17 balance and suppressed PI3K/AKT activation, the causal relationship between these two effects was not directly demonstrated. It remains unclear whether PI3K/AKT inhibition is the primary driver of immune rebalancing or merely a parallel outcome. Future studies using pathway-specific inhibitors or conditional knockouts could help establish causality. Second, it should be noted that the present study utilized exclusively male BALB/c mice. This choice was made primarily to minimize potential experimental variability associated with cyclical hormonal fluctuations, which can significantly influence immune responses. However, this approach inherently limits the generalizability of our findings to female populations or other genetic strains. Given that sex-based and strain-dependent differences are known to play critical roles in the pathogenesis and therapeutic response of allergic diseases [44], 45], future research incorporating female mice and diverse animal models is warranted to further validate the broad-spectrum efficacy and clinical translatability of Schis-A. Third, the current study primarily focused on the histological assessment of structural protection, such as epithelial thickness and goblet cell counts. While these results demonstrate significant mucosal improvement, specific molecular markers of the nasal barrier, including tight junction proteins (occludin and claudins) and upstream alarmins, were not evaluated. Given the critical role of barrier dysfunction in AR pathogenesis, future research focusing on these molecular components will be essential to fully elucidate the structural protective mechanisms of Schis-A. Additionally, this study focused on the acute phase of AR, and the 10-day treatment window precludes conclusions regarding the durability of Schis-A’s effects. Future research using chronic models and extended follow-up is necessary to evaluate long-term efficacy and the potential for sustained disease modification.

In summary, Schis-A alleviates AR symptoms and restores immune balance, potentially via PI3K/AKT pathway modulation.

## Supplementary Material

Supplementary Material

Supplementary Material

Supplementary Material

Supplementary Material
