# Supplementary material for: Schisandrin A alleviates allergic rhinitis by restoring Th1/Th2 and Treg/Th17 immune balance
Source: Open Life Sci. 2026 Aug 25;21(1):20251348. doi: 10.1515/biol-2025-1348 (PMC13527562; doi:10.1515/biol-2025-1348)
Supplement: Supplementary file 1 [file j_biol-2025-1348_suppl_001.docx]

**Supplementary Table S1. qRT-PCR primers used in the study**

| Gene symbol | Sense, 5’-3’ | Antisense,5’-3’ |
| --- | --- | --- |
| RORγt | CTCCATATTTGACTTTTCCCACT | GATGTTCCACTCTCCTCTTCTCT |
| Foxp3 | CGACCCCCTTTCACCTATGCC | TGCTCCCTTCTCGCTCTCCAC |
| β-actin | GGAGTACGATGAGTCCGGC | GTGTAAAACGCAGCTCAGTAACA |
